# Nutraceuticals as Alternative Approach against Cadmium-Induced Kidney Damage: A Narrative Review

**DOI:** 10.3390/metabo13060722

**Published:** 2023-06-02

**Authors:** Herbert Ryan Marini, Federica Bellone, Antonino Catalano, Giovanni Squadrito, Antonio Micali, Domenico Puzzolo, José Freni, Giovanni Pallio, Letteria Minutoli

**Affiliations:** 1 Department of Clinical and Experimental Medicine, University of Messina, 98125 Messina, Italy; federica.bellone@unime.it (F.B.); catalano.antonino@unime.it (A.C.); gsquadrito@unime.it (G.S.); giovanni.pallio@unime.it (G.P.); lminutoli@unime.it (L.M.); 2 Department of Human Pathology of Adult and Childhood, University of Messina, 98125 Messina, Italy; amicali@unime.it; 3 Department of Biomedical and Dental Sciences and Morphofunctional Imaging, University of Messina, 98125 Messina, Italy; puzzolo@unime.it (D.P.); jose.freni@unime.it (J.F.)

**Keywords:** cadmium, PTE, kidney, CKD, bone, oxidative stress, inflammation, apoptosis, microbiota, nutraceuticals, Mediterranean diet

## Abstract

Cadmium (Cd) represents a public health risk due to its non-biodegradability and long biological half-life. The main target of Cd is the kidney, where it accumulates. In the present narrative review, we assessed experimental and clinical data dealing with the mechanisms of kidney morphological and functional damage caused by Cd and the state of the art about possible therapeutic managements. Intriguingly, skeleton fragility related to Cd exposure has been demonstrated to be induced both by a direct Cd toxic effect on bone mineralization and by renal failure. Our team and other research groups studied the possible pathophysiological molecular pathways induced by Cd, such as lipid peroxidation, inflammation, programmed cell death, and hormonal kidney discrepancy, that, through further molecular crosstalk, trigger serious glomerular and tubular injury, leading to chronic kidney disease (CKD). Moreover, CKD is associated with the presence of dysbiosis, and the results of recent studies have confirmed the altered composition and functions of the gut microbial communities in CKD. Therefore, as recent knowledge demonstrates a strong connection between diet, food components, and CKD management, and also taking into account that gut microbiota are very sensitive to these biological factors and environmental pollutants, nutraceuticals, mainly present in foods typical of the Mediterranean diet, can be considered a safe therapeutic strategy in Cd-induced kidney damage and, accordingly, could help in the prevention and treatment of CKD.

## 1. Cadmium: Who, Where and How

Potentially toxic elements (PTEs) are defined as elements that can be found in water, soils, and sediments and are able to progressively accumulate and above certain limits to cause severe damage to humans, animals, and the environment [1,2]. They are classified as essential or nonessential elements [3]. The former are manganese, iron, nickel, and zinc, necessary for the processes of growth, development, and other physiological activities of the organism [4]. Nonessential elements, such as cadmium (Cd), arsenic, mercury, lead, etc., cause trouble in the biological activities of organisms [5], as they can accumulate in the body and are used as substitutes for essential elements. As an example, Cd is able to replace calcium, so that the normal bone structure is altered, inducing bone diseases (osteomalacia, decalcification, and osteoporosis) [6].

Cd is a PTE with atomic number 48, discovered in 1817 by Friedrich Stromeyer in some samples of zinc carbonate; its name, in fact, comes from the Latin word “cadmia” meaning calamine, a mix of minerals rich in zinc carbonate, or from the Greek word “kadmeia” with the same meaning [7].

Cd is particularly rare in the Earth’s crust, with a lithosphere concentration of about 0.1–0.2 mg/kg [8], but it can be mobilized into the atmosphere owing to the action of volcanoes and the weathering of rocks by wind and rain [9], causing pollution of environmental air.

From the atmosphere, Cd is released to agricultural soils in a quantity calculated at 2500–15,000 tons annually [10]. Once in the soil, Cd concentration can increase owing to the use of unprocessed drain waters, phosphate-based fertilizers, particularly those obtained from seabed sediments with high Cd content [10], or different anthropogenic activities. Cd is easily absorbed by plants owing to its high mobility within the soil-plant system [11], even if its uptake is regulated by many factors related to soil characteristics, for example particle size, pH, temperature, and plant activity, such as root size and rate of root exudation and transpiration [12]. Cd absorption from plants may result in serious health problems. In fact, evidence was provided that in people living in Cd-uncontaminated locations, Cd-containing foods, such as vegetables, cereals, and legumes, are the main source of Cd either in animals or in humans [10]. It must be kept in mind that Cd is toxic to humans at lower concentrations than plants; therefore, apparently healthy plants are not safe for human feeding [10].

Anthropogenic activities are considered to contribute almost 80–90% of Cd pollution in the environment [13]. In fact, in addition to the use of phosphate fertilizers because of incorrect waste management, a higher concentration of Cd in the soil was observed around mining areas and industries where Cd is used for many purposes [3]. The extraction of minerals, even if crucial for human progress, causes serious PTE pollution in the environment [14]. Owing to its peculiar characteristics, including great electrical conductivity, resistance to corrosion, and low melting point, Cd has many industrial usaes, including anticorrosive materials production, electronic constituents, plastic stabilizers, nickel-Cd batteries, paints, and pigments [6].

Once in the environment, Cd is available for absorption by the organism. In humans, different ways of penetration have been described: the respiratory apparatus, the digestive apparatus, and the skin.

The main route of Cd exposure is considered cigarette smoke; in fact, Cd is accumulated by tobacco plants in a high concentration (650 to 3630 ng/g tobacco) [15], particularly when they are grown in contaminated soils. Another important environmental respiratory entry is found in workers of mines, and in factories producing paints and batteries, owing to the noteworthy quantities of Cd contained in dust and fumes [16]. It was demonstrated that particles containing Cd are able to induce a direct noxious effect on both cell types (type I and type II pneumocytes) of the alveolar epithelium with cellular injury, inflammation, and fibrosis, increasing the possibility of respiratory diseases [17,18,19]. A large quantity of Cd (50–100% of the inhaled particles with diameters smaller than 2–3 μm) [17] is entrapped in the epithelium, crosses the pulmonary interstice and enters the circulation.

In nonsmokers, food is the main cause of Cd intake, and its absorption is related to the type of toxicant, the amount, and the rate of exposure [20]. Cell death after chronic Cd exposure may cause structural changes of the intestinal epithelium, resulting in larger amounts of Cd permeation. Similarly, Cd-induced lesions of epithelial tight junctions may allow further penetration of Cd through the intestinal barrier [21]. However, it was recently demonstrated that gut bacteria can decrease the intestinal permeability of Cd, thus providing direct protection of the barrier [22]. From the epithelium, Cd is absorbed into the connective tissue and then into the submucosal capillaries.

As to skin absorption, previous experimental papers demonstrated the accumulation of Cd in the shaved skin of mice and rats, causing hyperkeratosis, acanthosis, and ulcerative changes in a dose-related manner [23]. The role of Cd in the prevention of skin pathologies was recently shown in psoriatic subjects evaluated in the NHANES study, which demonstrated a correlation between blood Cd and psoriasis severity [24].

**Figure 1:**
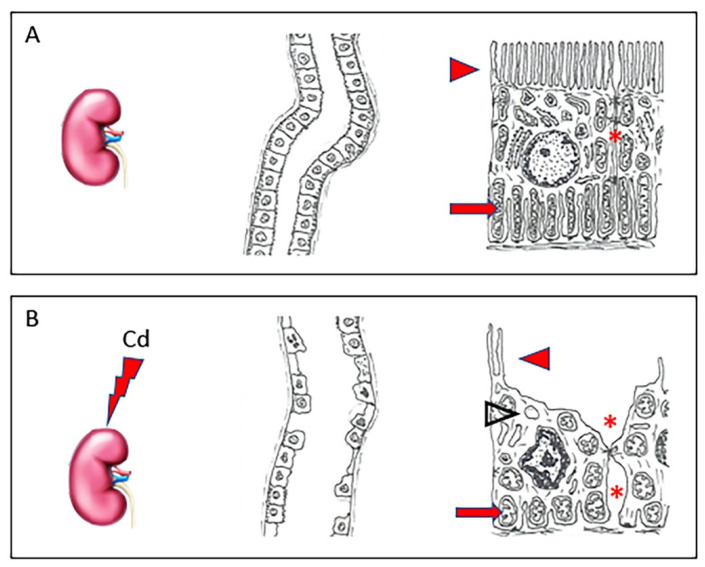
In a healthy kidney (**A**), tubules have normal architecture; their epithelium shows well-evident apical microvilli (arrowhead), elongated mitochondria (arrow), and tight intercellular junctions (asterisk). In kidneys challenged with Cd (**B**), tubules have evident morphological changes, as epithelial cells show short, few, or even absent apical microvilli (arrowhead), round or swollen mitochondria (arrow), and cytoplasmic vacuoles (empty arrowhead). Intercellular spaces are wide (asterisk).

In the circulation, about 90% of Cd binds to α2-macroglobulin and albumin in the serum [25] and reaches the liver, where the complex is destroyed and small cysteine-rich proteins, metallothioneins (MT), are produced [26]. Of the four main isoforms (MT-1, -2, -3, and -4), Cd, likewise to other PTEs, induces the synthesis of MT-1 and MT-2, able to stimulate specific transcriptional factors, while MT-3 and MT-4 seem to have no role in the detoxification of PTEs [27]. The complex Cd-MT is taken from hepatocytes to shield the cells from toxic Cd ions. The excess part of these complexes not stored in the liver is discharged into the blood, reaching the kidney [16], where they are filtered from the glomerulus and then reabsorbed by the proximal tubular epithelial cells [28]. Here, the complex Cd-MT is degraded by lysosomes into amino acids and free Cd ions. In this way, free Cd can accumulate and cause nephrotoxicity, primarily in the proximal tubular region [29]. In fact, as demonstrated by our group in a recent paper [30], in healthy kidneys, tubules have an epithelium with well-evident apical microvilli, elongated mitochondria, and tight intercellular junctions. On the contrary, in kidneys challenged with Cd, tubules show evident morphological changes, as epithelial cells have short, few, or even absent apical microvilli, round or swollen mitochondria, and cytoplasmic vacuoles. Intercellular spaces are wide (Figure 1).

In the last few years, it has been focused on the relative roles of apoptotic, necrotic, and autophagic mechanisms in Cd-induced proximal tubular cell death. So far, these studies have implicated, from a pathophysiological point of view, three possible early response mechanisms in the proximal tubule. These are: (i) disruption of cadherin-mediated cell-cell adhesion; (ii) modulation of intracellular signaling cascades; and (iii) induction of oxidative stress [31]. This discovery has crucial implications for biomonitoring Cd-exposed populations and for the potential treatment of Cd nephrotoxicity. In this context, one novel marker that has shown exceptional promise in preclinical studies is Kidney Injury Molecule-1 (KIM-1). KIM-1 is a transmembrane protein that is not detectable in normal kidney but is expressed at high levels in the proximal tubule after ischemic or toxic injury [32]. Overall, these findings, along with early detection with novel biomarkers such as KIM-1, suggest that it may be possible to use specific agents to modulate or even halt these pathophysiological processes before they become irreversible [31].

The toxic action of Cd was also demonstrated in glomeruli, which showed elongated and fewer podocytes with reduced or lacking contact with the capillaries [30].

Recent data suggest that up to 50% of the deposits of Cd are accumulated in the kidney [28], where it induces renal toxicity owing to its mean half-life. Even if it were stated that the mean half-life of Cd in the kidney is 14 years [33], many variations ranging from 9 to 45 years and correlated with individual variations of MT expression were described [34]. Cd-induced renal toxicity is, therefore, a major risk for human health, particularly in countries where environmental controls are lacking. In fact, in patients exposed to Cd, a significant proximal tubular dysfunction was described, clinically expressed as increased urinary excretion of low-molecular-weight proteins, glucose, amino acids, and electrolytes such as sodium, potassium, and calcium [34]. Several studies have shown that Cd exposure may be related to chronic kidney disease (CKD) [35,36,37]. However, it is not easy to find a reliable exposure biomarker since several studies showed differences in study design (i.e., cross-sectional design) and/or exposure levels. To date, dietary urinary Cd (UCd) or blood Cd (BCd) have been commonly adopted as exposure biomarkers, as have urinary N-acetyl-β-d-glucosaminidase and beta-2-microglobulin. Generally, BCd mainly reflects recent exposure [38], while UCd may be related to long-term exposure [39,40]. Dietary Cd intake is also used as a surrogate indicator of Cd exposure [41]. Finally, Kawata [42] indicated that renal tubular function should be controlled during analysis. Overall, longitudinal studies are needed to better clarify the link between CKD and biomarkers.

## 2. Nutraceuticals: Generalities

The word “nutraceutical” (a combination of the terms “nutrient” and “pharmaceutical”) refers to “foods (or part of a food) that provide medical or health benefits, including prevention and/or treatment of disease” [43]. Nutraceuticals and pharmaceuticals exhibit high similarities and overlaps among their properties and functionalities [44]. To date, three groups of “healthy foods (or part of a food)” are considered: (i) “dietary supplements”, (ii) “functional foods”, and (iii) “nutraceuticals”. These latter may range from isolated nutrients, herbal products, dietary supplements, novel foods, and processed food ingredients. Indeed, in the global marketplace, nutraceuticals have become a multibillion-dollar industry as consumers in different countries appreciate these substances owing to their plant origin [45]. The popularity of nutraceuticals is also associated with their easy availability, low cost, and their intake in low doses. For this reason, the use of nutraceuticals in the prevention of renal dysfunction and CKD is a very intriguing option [46]. However, for many products, there is no clear data on their safety and effectiveness, possible side effects, interactions with prescribed medicines, or impact on preexisting medical conditions. Moreover, some nutraceuticals may present toxicity and cause adverse interactions with drugs commonly prescribed for CKD [47]. Overall, the topic is debated, and current research would help understand if it will be possible to employ “nutraceuticals” as an alternative approach against Cd-induced kidney damage.

## 3. Cadmium-Induced Pathophysiological Mechanisms and Kidney Dysfunctionality

Kidney damage induced by Cd has been shown either in vitro or in vivo [48,49,50]. In Japan, the Itai-itai disease, which is able to cause typical signs of CKD such as proteinuria, glicosuria, and aminoaciduria progressively irreversible, was shown to be related to chronic Cd toxication [51,52,53]. Generally, the above-mentioned features are typical of either occupational or environmental Cd poisoning, as experimentally observed. Cd exposure can also impair calcium metabolism, causing hypercalciuria and the formation of kidney stones [54]. The negative molecular cascade is amplified by the generation of reactive oxygen species (ROS), which are able to cause programmed cell death [55]. ROS in turn cause lipid peroxidation and damage to proteins, including Na+/K+ ATPase [56].

Oxidative stress can also lead to inflammation with increased production of proinflammatory cytokines, various chemokines, cellular adhesion molecules, and inducible enzymes that in turn can contribute to CKD [57,58,59,60,61]. In fact, the ROS increase induced by Cd challenge activates nuclear factor kappa B (NF-κB), which is a transcription factor able to control inflammation and regulate some components of the immune system. Once induced, it moves into the nucleus, regulating the synthesis of different mediators, such as tumor necrosis factor (TNF)-α, interleukin (IL)-6, IL-12, cycloxygenase-2 (COX-2), inducible nitric oxide synthase (iNOS), and macrophage migration inhibitory factor [62,63]. Therefore, inflammatory and immune disorders could be the consequence of NF-κB dysregulation; moreover, as reported in their intriguing review, Satarug and coworkers [64] highlighted the role of inflammation and oxidative stress as mechanistic pathways altered by Cd exposure (“the perfect storm”) in the physiopathology of diabetes and hypertension, that, in turn, cause CKD.

Additionally, Cd-induced renal inflammation through the NF-κB signaling pathway is able to activate the NLR family Pyrin Domain Containing 3 (NLRP3) inflammasome, a component of the innate immune system, whose role is still to be fully elucidated [65].

Lipid peroxidation, in turn, induces apoptosis by the following mechanisms: (i) the endoplasmic reticulum (ER)-mediated pathway through ER stress and calcium release; (ii) the mitochondria-mediated molecular signals; and (iii) the p53-dependent apoptotic pathway [50].

Specifically, it has been shown that the Cd-induced oxidative stress/inflammatory cascade activates apoptosis through the Fas/FasL pathway [66], and this molecular signal appears crucial in CKD induced by different nephrotoxic agents [67,68,69]. In the kidney, as also observed by our research group, a crucial role for mitochondria-dependent apoptosis is played by the B-cell lymphoma-2 (Bcl-2)/Bcl-2-associated X protein (Bax) system [70]. Moreover, it has been demonstrated in kidney tubules that, after Cd challenge, autophagia followed by apoptosis involves the upregulation of KIM-1 expression and changes in the localization and function of typical transmembrane adhesion molecules such as N-cadherin and claudin-2 [71,72]. The reduction of N-cadherin and claudin-2, a which are able to modify tubular epithelial polarization and junctional complexes, can be related to the presence of KIM-1 [31]. Finally, it was demonstrated that, experimentally, Cd suppressed renal erythropoietin (EPO) production through a direct effect and destruction of EPO-producing cells, driving anemia in Cd toxicity [73]. Moreover, it has been suggested that inhibition of EPO gene expression by Cd depends on the suppression of Hypoxia-Inducible Factor (HIF)-1 binding activity [74].

## 4. Cadmium and Bone Damage in CKD

**Figure 2:**
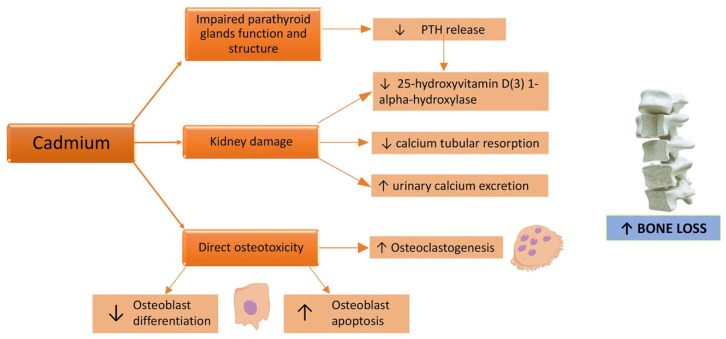
Effects of a cadmium challenge on the bone. ↑: increased; ↓: decreased.

Osteotoxicity is a known effect of Cd [75]. In fact, the Itai-Itai disease, caused by a chronic exposure to Cd due to the use of Cd-polluted water to irrigate the rice fields [38], in addition to kidney failure, caused osteomalacia and osteoporosis. Skeleton fragility related to Cd exposure can be induced both by a direct Cd toxic effect on bone mineralization and by renal failure (Figure 2), even if the critical exposure levels and exact underlying mechanisms remain unclear [76].

Cd exerts its direct toxicity either by a reduction in bone formation or an increase in bone resorption [77]. Indeed, this PTE affects mostly osteoblastic cells via the inhibition of osteoblast differentiation, synthesis activity, and the mineralization process of the extracellular matrix [78]. Scimeca et al. analyzed bone head biopsies demonstrating that Cd accumulation was associated with lower bone quality parameters and reduction and/or absence of osteoblasts; curiously, through an immunohistochemistry method, high levels of sclerostin, a glycoprotein belonging to the family of bone morphogenetic protein antagonists, were found in bone tissue of osteoporotic patients with Cd accumulation [79]. Cd exposure also determines an increase of tartrate resistant acid phosphatase (TRAP) activity and the formation of TRAP positive activated osteoclasts in the presence of receptor-activated nuclear factor κ B ligand (RANKL), inducing the differentiation of osteoclast precursors into osteoclasts and consequently leading to increased bone resorption [80].

Furthermore, Cd could induce proximal tubular dysfunction [77] with impaired calcium tubular resorption and consequently augmented urinary calcium excretion [81]; this, in turn, leads to bone demineralization and an increased risk of kidney stones [82]. Interference with parathyroid hormone (PTH) release has also been demonstrated: in the setting of Cd exposed workers, a significant negative correlation between the Cd-exposure index and plasma PTH levels was shown [83]. In other terms, Cd exposure leads to a decrease in PTH levels [84]. A structural and functional damage of the parathyroid glands, with a dose-dependent behavior and intensity related to Cd exposure duration has been demonstrated in murine models [85]. This implies a reduced activation of 25(OH)D3 vitamin D, because of impaired 1-alpha-hydroxylation in the kidney from its inactive form to the active one, 1,25(OH)2D3, followed by reduced intestinal calcium absorption and decreased reabsorption of bone mineral matrix [54]. Many cross-sectional and prospective population-based studies showed a negative correlation between Cd exposure and bone mineral density (BMD) [86,87,88,89,90,91,92] (Table 1).

In detail, U-Cd concentrations have been contrariwise associated with BMD at the total body, lumbar spine, hip, femoral neck, and volumetric femoral neck [93]. Nevertheless, Kim et al. recently demonstrated no direct dose-response relationship at the highest Cd levels; this was related to a greater awareness of the disease by participants with osteoporosis and to higher Cd levels, resulting in improved therapeutic adherence, resulting in better BMD. Another reason for their results could simply be selection bias [92].

To date, little evidence exists on the protective role of some nutraceuticals against the damage to bone integrity induced by Cd exposure [94]. The supplementation of a natural polyphenol, resveratrol (RES), was shown to prevent Cd-induced apoptosis in osteoblastic MC3T3-E1 cells and to mitigate the inhibition of osteogenic differentiation induced by Cd chloride (CdCl_2_) by modulating ERK1/2 and JNK signaling [95]. Zinc supplementation has been demonstrated to prevent an increased risk of femoral neck fractures in rats with chronic exposure to Cd [96]. In Cd-exposed rats with a vitamin D-deficient diet, the toxic effect of Cd on kidney, bone, and hematopoietic systems was significantly higher than in Cd-exposed rats with a normal diet, suggesting a potential protective role of vitamin D administration against Cd-induced bone and kidney damage [54]. Through its antioxidant activity, spirulina, a filamentous cyanobacterium (also called blue-green algae), showed a significantly reduced frequency of fetal anencephaly, micro maxillary deformity, and skeletal deformities in pregnant mice orally administered with a high dose of Cd [97]. Essential elements, such as calcium, zinc, and vitamins, with which Cd shares a very similar way of metabolism and absorption, can attenuate Cd toxicity, particularly in bone tissue [94].

Notwithstanding, further research is needed to better define dietary strategies for preventing Cd-induced bone loss.

## 5. Therapeutic Effects of Functional Foods and Nutraceuticals in Cadmium-Induced Kidney Dysfunctionality: The Latest Preclinical Updates

**Figure 3:**
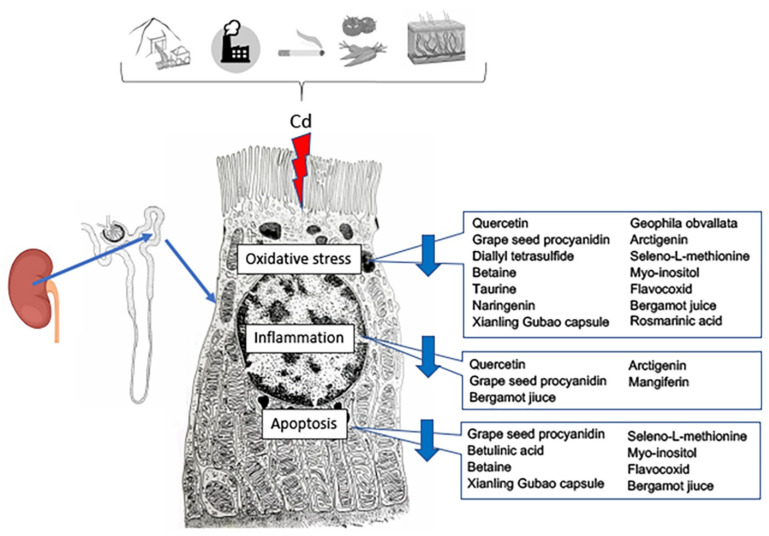
Effects of nutraceutical treatment on the different pathways triggered by cadmium challenge in the proximal tubule cells of the kidney.

In the last few years, the protective role of antioxidants in food against PTEs has been evaluated [98] (Table 2; Figure 3).

In fruits, vegetables, and wine, the polyphenolic compounds, flavonoids, are broadly distributed. Specifically, quercetin is the most abundant (60–75% of the polyphenols ingested). Quercetin has antioxidant, anti-inflammatory, and chelating activities, so it is protected from nephrotoxicity even after Cd intoxication [63,99]. It acts mainly as an antioxidant by contrasting the action of superoxide anion and lowering xanthine, NADPH oxidase, and superoxide dismutase (SOD). Moreover, an indirect action through increased MT-1 and MT-2 activity has been revealed [63,99]. In fact, the administration of quercetin plus Cd has increased MT-1 and MT-2 expression, thus lowering acute renal Cd toxicity, probably owing to its antioxidant activity. Quercetin also shows a crucial anti-inflammatory action through an augmented activity of both MT and endothelial nitric oxide synthase (eNOS) expression, together with an inhibition of both COX-2 and iNOS expression. Finally, a potent chelating capacity of quercetin, through the reduction of Cd uptake and accumulation in the kidney, has been demonstrated to further protect against Cd tubular damage [63,99].

Another substance with widespread antioxidant and anti-inflammatory action is grape seed procyanidin extract (GSPE), which is typical of tea leaves, fruits, vegetables, and seeds of many plants, such as grapes and apples [100]. When compared to vitamins C, E, and β-carotene, GSPE demonstrated a broad spectrum of antioxidant activity [101]. In Cd-challenged mice, GSPE was able to increase glutathione (GSH)-peroxidase (GPx) and SOD activities and decrease malondialdehyde levels in the kidneys. Moreover, GSPE antagonized renal apoptosis, as indicated by the expression of Bax and Bcl-2 [100].

Betulinic acid, a natural pentacyclic triterpenoid present in the bark of a number of trees, including white birch, bear tree, sycamore, and other members of the Platanus family, has, among other things, antioxidative, anti-inflammatory, and anti-apoptoptic properties. A protective effect of betulinic acid in the course of renal ischemia/reperfusion was demonstrated, as it induced antioxidant responses, improved structural changes, and renal function by modulating apoptosis of leukocytes [116]. Recently, a positive effect of betulinic acid on CdCl_2_-induced kidney injury was demonstrated by a direct inhibition of apoptosis [102].

Diallyl tetrasulfide (DTS) is a substance with antioxidant effects, found in garlic and, as an essential oil, in other plants [103]. It protects tubular cells, either in vivo or *in vitro*, after CdCl_2_ toxicity, owing to its antioxidant and metal chelating activities [48].

Betaine (glycine betaine or trimethylglycine), a natural antioxidant, can be obtained from the diet or from its precursor, choline [117]. As a result, reduced lipid peroxidation, an increased antioxidant status, a blunting of caspase-3 activity, and a reduction of tubular morphological changes were observed in the kidneys of rats challenged with Cd plus betaine [104].

An evident protection against oxidative stress caused by a Cd challenge was also observed after administration of the essential amino acid taurine (2-aminoethanesulfonic acid) [105,106]. When taurine is administered before Cd challenge, a reduction of morphological damages and of antioxidant enzyme levels, such as catalase (CAT), glutathione S-transferase (GST), glutathione reductase (GR), SOD, GPx, and glucose-6-phosphate dehydrogenase, was observed in mice’s kidneys [105].

Another substance with strong nephroprotective activity (antioxidant and metal chelating properties) is the bioflavonoid naringenin (4,5,7-trihydroxy flavonone), particularly abundant in citrus fruits [49,107]. A significant reduction of the structural changes and an increase of antioxidants and glutathione metabolizing enzymes were observed in the kidneys of Cd-exposed rats after oral coadministration of naringenin [49].

Xianling Gubao Capsule, a preparation of a mixture of Chinese herbs [Epimedii Folium (Epimedium brevicomu Maxim), Salvia miltiorrhiza Radix Rhizoma (Salvia miltiorrhiza Bunge), Anemarrhenae Rhizoma (Anemarrhena asphodeloides Bunge), Psoraleae Fructus (Cullen corylifolium (Linnaeus) Medikus), Dipsaci Radix (Dipsacus asper Wallich ex Candolle), and Rehmanniae Radix (Rehmannia glutinosa Libosch. ex Fisch. et Mey)] showed an important protective role in Cd-exposed mice, as it positively regulated oxidative stress, autophagy, and apoptosis, owing to the actions of the single components of the mixture [108].

The methanolic extract of Geophila obvallata (Rubiacea), a medicinal herb used in African ethnomedicine for treating kidney diseases, possesses bioactive principles able to show potent antioxidant action and downregulate KIM-1 and MT-1 in rats, thus providing renal protection against Cd-induced nephrotoxicity [109].

Arctigenin, a lignan naturally present in several plants, showed anti-inflammatory and antioxidant actions and reduced the expression of KIM-1 in the kidneys of Cd-treated rats [110].

Mangiferin (MGN) is a glucosylxanthone particularly abundant in the leaves and edible mango fruits of Mangifera indica. In vitro studies showed that MGN showed a potent antiinflammatory effect against Cd toxicity in human glomerulus renal endothelial cells through the reduction of IL-6 and IL-8, which play a significant role in renal inflammation [111].

Rosmarinic acid (RA), a naturally occurring polyphenolic nutraceutical, is an active constituent of Rosmarinus officinalis. In vitro and in vivo data revealed that RA treatment significantly counteracted the Cd-induced nephrotoxicity by blunting ROS, promoting cellular redox defense, and Cd clearance, thus positively modulating the altered pathological signal transduction [112].

An evident protection from oxidative stress of the kidney both in vivo and in vitro was observed after treatment with selenium (Se), which was related to ROS scavenging [113,114], through the activation of c-Jun N-terminal kinase phosphorylation [115]. Se inhibited the oxidative stress based on a reduction of ROS and blunted apoptosis through mitochondrial dysfunction, then confirmed a cytoprotective role against Cd toxicity in the kidney [113,114].

The treatment with the natural nutraceutical myo-inositol (MI) in Cd-treated mice showed protection against kidney damage. In fact, MI significantly reduced urea nitrogen and creatinine levels, oxidative marker expression, modulated apoptosis, increased GSH content and GPx activity, and preserved kidney morphology, suggesting a strong antioxidant role against Cd with harmful effects on kidney lesions [72].

Flavocoxid, a flavonoid containing both baicalin from Scutellaria baicalensis (Chinese skullcap) and catechin from Acacia catechu (Black catechu), reduced CdCl_2_-induced oxidative damage secondary to ROS generation in the kidney of C57 BL/6J mice. A significant reduction of iNOS, phosphoextracellular signal-regulated protein kinase 1/2, and matrix metalloproteinase-9 expression and of morphological changes of glomeruli and proximal tubules was in fact observed [30].

Recently, our research group evaluated the effects of a flavonoid-rich extract of bergamot juice (BJe), alone or in association with curcumin and resveratrol, in the kidneys of mice exposed to CdCl_2_ [70]. BJe, obtained from Citrus bergamia Risso et Poiteau (bergamot) fruits, showed antioxidant, anti-inflammatory, and antiapoptotic properties, as it significantly decreased urea nitrogen and creatinine levels, along with p53, Bax, Nos2, and IL-1ß mRNA, while increasing Bcl2, glutathione content, and glutathione peroxidase activity. Moreover, there was also a reduction of the glomerular and tubular damage, and of nuclear factor erythroid factor 2-related factor 2, NAD(P)H:quinone acceptor oxidoreductase 1 and heme oxygenase 1 gene expression, thus suggesting a new potential strategy in the management of CKD in subjects exposed to environmental toxicants.

## 6. Nutraceuticals and Microbioma: Putative Role in Cadmium-Induced Kidney Damage

The importance of the complex interactions between the microbiome and the human body is now well recognized, and the contributions of this relationship to host health are increasingly appreciated [118].

Indeed, any discussion of functional foods, nutraceuticals, or dietary supplements in the context of PTE-induced organ damage should address the impact on the microbiome of food and all potential interactions with a preventive and/or therapeutic intervention [119].

Several nutraceuticals act as “prebiotics”, which according to a description by a panel of experts convened by the International Scientific Association for Probiotics and Prebiotics (ISAPP), are defined as “a substrate that is selectively utilized by host microorganisms conferring health benefit” [120]. In this context, the metabolic products of the microbiota, such as short-chain fatty acids (SCFAs) and gases [121,122], appear to play a crucial role in the host.

Therefore, CKD is associated with the presence of dysbiosis, and the results of recent studies have confirmed the altered composition and functions of the gut microbial communities in CKD. In fact, during CKD, protein-bound uremic toxins are progressively accumulated [123]. Moreover, the presence of CKD may be accompanied by the development of intestinal inflammation and epithelial barrier impairment, leading to the translocation of bacterial-derived uremic toxins to the submucosal compartment, where they activate mast cells and lymphocytes, causing the release of proteases, cytokines/chemokines, and other crucial mediators of inflammation. In other words, the loss of kidney function results in structural and functional alterations of the intestinal barrier, contributing to the syndrome of uremia. This finding strongly suggests that a complex bidirectional metabolic and immunological crosstalk involving the kidney and gut is present [124]. Moreover, the aforementioned molecules can activate sensory afferents leading to local reflex responses and/or central transmission, as well as gain access to the portal and systemic circulations via the submucosal vasculature, leading, in turn, to oxidative stress injury, particularly involving the cardiovascular and endocrine systems [125].

Recently, it has been suggested that one of the useful properties of probiotic bacteria is their capacity to bind different targets, thus eliminating them through feces [126]. Specifically, it is supposed that one of these targets could be Cd. As a matter of fact, Djurasevic and coworkers experimentally showed that the rise in lactobacilli number in the feces of rats treated simultaneously with Cd and probiotics resulted in a strong correlation between the increase in Cd concentration in their feces and the decrease in Cd concentration in their blood. These findings suggest that probiotics actively contribute to Cd excretion through feces, probably by binding to the bacterial cell wall, opening the possibility of their therapeutic applications against Cd toxicity [126].

So far, gut microbiota are very sensitive to nutraceuticals, functional foods, probiotics, diet, and even environmental pollutants. Then, it is undeniable that dietary components and supplements interact in one way or another with the gut microbiome. Therefore, the possible effects on the health of environmental pollutants such as antibiotics, PTEs (including Cd), persistent organic pollutants, pesticides, nanomaterials, and food additives on the gut microbiota and their subsequent effects will continue to represent a major focus of future experimental [127] and clinical research [128].

## 7. Nutraceuticals and CKD: Chances and Limits in Routine Clinical Setting

Currently, there is no effective treatment for Cd poisoning. The principal therapeutic protocol involves the employment of metal chelators, although they cause several undesirable effects, such as redistribution/translocation of PTEs and other serious toxic events. This caught the interest of scientists who have sought an effective remedy from natural sources and/or from foods/healthy eating habits that are less likely to produce toxic effects. In this context, it appears crucial to add more information on the molecular mechanisms of Cd-induced structural damage of the kidney leading to CKD. So far, although in the present narrative review we considered a lot of preclinical studies and new data are currently available, unfortunately, to date, it is very hard to define Cd exposure levels related to the described biological effects on the kidney and the overall human health risk assessment. However, despite this scarcity of information and the limitations related to the type of review, dietary strategies and the use of nutraceuticals, which are present in foods typical of Mediterranean-style eating patterns, appear very useful in the management of non-communicable diseases, particularly CKD [129,130,131]. Pérez-Torres and colleagues, in their recent review [131], suggest a practical approach to Mediterranean diet adaptation as nutritional treatment in CKD patients. Indeed, there are several studies that suggest the use of a Mediterranean-style eating pattern as the dietary approach of choice for patients with CKD, regardless of the CKD stage [132,133]. In this context, it is well-known that the traditional Mediterranean diet is particularly abundant in cereals, legumes, nuts, fruits, vegetables, and herbs, and low in red meat [134]. Moreover, this dietary pattern includes a moderate intake of fish, seafood, eggs, white meat, and dairy products, and a moderate intake of alcohol (mainly red wine); finally, extra virgin olive oil is the main source of added fat [134]. So far, foods typical of Mediterranean-style eating patterns and the related compounds are well-known and under current careful investigation by several research groups for their potential benefit in positively modulating of the endothelial function, inflammation, oxidative stress, lipid profile, and blood pressure, that are crucial risk factors for the development of non-communicable diseases, including CKD [135,136]. Of course, in this context it should be carefully focused on a synergistic or antagonistic action between different bioactive foods or nutraceuticals of the Mediterranean-style eating pattern and, more generally, in plant-based diets, on neuroendocrine immune system modulation and gut microbiota dysbiosis, even more so in the presence of environmental pollution [129,130] caused by PTEs as Cd.

## 8. Conclusions

The molecular mechanisms of Cd-induced structural and functional damage to the kidney are a research topic of current interest. Experimental and clinical data analyzed in the present narrative review suggest that the multifaceted mechanism of action of nutraceuticals needs to be taken serious to effectively counteract the detrimental molecular cascade in kidney injury caused by environmental PTEs such as Cd.

## Figures and Tables

**Table 1:** Main clinical studies about cadmium exposure and fracture risk.

Authors	Study Design	Population	Measurements	Main Findings
Alfvén et al., 2000 [86]	Retrospective cohort study	520 men and 544 women, aged 16–81 years, environmentally or occupationally exposed to Cd for at least 5 years	U-Cd, protein HC forearm BMD by DXA	U-Cd was negatively related to BMD, particularly in patients aged more than 60; in men over 60 the ORs for osteoporosis in the highest U-Cd category were 3.5 (95% CI, 0.6–19) in the group without tubular proteinuria, and 4.2 (95% CI, 1.0–20) in the group with tubular proteinuria
Engström et al., 2012 [87]	Prospective cohort study	2676 women aged 56–69 years selected from the Swedish Mammography Cohort	Dietary Cd exposure assessed by a food frequency questionnaire, U-Cd BMD at the total body, femoral neck and lumbar spine by DXA, incidence of fractures	High dietary Cd exposure (≥13 μg/day, median) was associated with an increased risk of osteoporosis (OR = 1.32; 95% CI: 1.02–1.71) and of any first incident fracture (OR = 1.31; 95% CI: 1.02–1.69)
Chen et al., 2014 [88]	Cross-sectional, case–control study	321 Chinese subjects (202 women and 119 men), aged 27 years and older living in control and polluted areas	U-Cd, U-Pb, B-Cd and B-Pb BMD at the proximal radius and ulna by DXA	Cd and Pb levels of people in the polluted area higher than those in the control area (*p* < 0.05); BMD of women in the polluted area lower than that of women in the control area (*p* < 0.05) and BMD decreased with increasing of B-Cd (*p* < 0.05), B-Pb and U-Pb in women. The likelihood of low BMD was associated with higher B-Cd in women (OR = 2.5, 95% CI: 1.11–5.43) and B-Pb in men (OR = 4.49, 95% CI: 1.37–14.6)
Lim et al., 2016 [89]	Nationwide cross-sectional study	Data of 2429 subjects from the KNHANES between 2008–2011	B-Cd, B-Pb and B-Hg BMD at total hip, femoral neck and lumbar spine	In subjects with the highest quartile of B-Cd (≥1.439 μg/L) the risk for osteopenia or osteoporosis increased 2.1 times (95% CI 1.64–2.68)
Wallin et al., 2016 [90]	Prospective cohort study	936 men from the MrOS study aged 70 to 81 years	U-Cd BMD at total body, hip, and lumbar spine, incidence of fractures	Significant negative associations between U-Cd and BMD, with lower BMD (4% to 8%) for all sites in the fourth quartile of U-Cd; positive associations between U-Cd and incident fractures, especially nonvertebral fractures in the fourth quartile of U-Cd
Lv et al., 2017 [91]	Cross-sectional study	1116 subjects (832 and 284 subjects from a Cd-polluted area and a non-Cd-polluted area respectively)	U-Cd BMD at forearm	Significant negative association of U-Cd concentrations with BMD
Kim et al., 2021 [92]	Nationwide cross-sectional study	Data of 1031 post-menopausal women ≥50 years of age from the 4th and 5th KNHANES	B-Cd, nutrient intake BMD at total hip, femoral neck, and lumbar spine by DXA	Significant positive association between B-Cd levels and the risk of osteopenia and osteoporosis, but the OR at the 4th level was lower than that at the 3rd level (OR and 95% CI for osteopenia: 2nd quartile: 1.24, 0.88–1.74; 3rd quartile: 3.22, 2.24–4.64; 4th quartile: 1.27, 0.87–1.85; *p* < 0.001; OR and 95% CI for osteoporosis: 2nd quartile: 1.54, 1.05–2.25; 3rd quartile: 3.63, 2.31–5.69; 4th quartile: 1.70, 1.03–2.81; *p* < 0.001)

**Table 2:** Data obtained from in vivo and in vitro studies on nutraceuticals utilized as a possible approach against Cd-induced kidney toxicity.

Authors	Study Design	Sample	Substance
Morales AI et al., 2006 [63] Morales AI et al., 2006 [99]	In vivo	Rats	Quercetin
Bagchi D et al., 2002 [100] Chen Q et al., 2013 [101]	In vitro In vivo	Human cells Mice	Grape seed procyanidin extract (GSPE)
Fan R et al., 2018 [102]	In vivo	Mice	Betulinic acid
Pari L and Murugavel P, 2005 [103]	In vivo	Rats	Diallyl tetrasulfide (DTS)
Hagar H and Al Malki W, 2014 [104]	In vivo	Rats	Betaine
Hwang DF and Wang LC, 2001 [105] Manna P et al., 2009 [106]	In vivo	Rats Mice	Taurine
Verma S et al., 2021 [107]	In vivo	Fish	Naringenin
Huang J et al., 2021 [108]	In vivo	Mice	Xianling Gubao
Iserhienrhien LO and Okolie NP, 2021 [109]	In vivo	Rats	Geophila obvallata
Salama SA et al., 2021 [110]	In vivo	Rats	Arctigenin
Rajendran P et al., 2016 [111]	In vitro	Human cells	Mangiferin
Joardar S et al., 2019 [112]	In vitro	Murine kidney cells	Rosmarinic acid
Wang Y et al., 2013 [113] Zhou YJ et al., 2009 [114] Shi Q et al., 2019 [115]	In vitro In vivo	LLC-PK1 cells Chicken	Selenium (Se)
Pallio G et al., 2019 [72]	In vivo	Mice	Myo-inositol
Micali A et al., 2018 [30]	In vivo	Mice	Flavocoxid
Cirmi S et al., 2021 [70]	In vivo	Mice	Bergamot juice extract (BJe)
